# Alpha and theta oscillations on a visual strategic processing task in age-related hearing loss

**DOI:** 10.3389/fnins.2024.1382613

**Published:** 2024-07-16

**Authors:** Shraddha A. Shende, Sarah E. Jones, Raksha A. Mudar

**Affiliations:** ^1^Department of Communication Sciences and Disorders, Illinois State University, Normal, IL, United States; ^2^Department of Speech and Hearing Science, University of Illinois Urbana-Champaign, Champaign, IL, United States

**Keywords:** age-related hearing loss, strategic processing, cognitive control, alpha, theta, neural oscillations, older adults, event-related electroencephalography

## Abstract

**Introduction:**

Emerging evidence suggests changes in several cognitive control processes in individuals with age-related hearing loss (ARHL). However, value-directed strategic processing, which involves selectively processing salient information based on high value, has been relatively unexplored in ARHL. Our previous work has shown behavioral changes in strategic processing in individuals with ARHL. The current study examined event-related alpha and theta oscillations linked to a visual, value-directed strategic processing task in 19 individuals with mild untreated ARHL and 17 normal hearing controls of comparable age and education.

**Methods:**

Five unique word lists were presented where words were assigned high- or low-value based on the letter case, and electroencephalography (EEG) data was recorded during task performance.

**Results:**

The main effect of the group was observed in early time periods. Specifically, greater theta synchronization was seen in the ARHL group relative to the control group. Interaction between group and value was observed at later time points, with greater theta synchronization for high- versus low-value information in those with ARHL.

**Discussion:**

Our findings provide evidence for oscillatory changes tied to a visual task of value-directed strategic processing in individuals with mild untreated ARHL. This points towards modality-independent neurophysiological changes in cognitive control in individuals with mild degrees of ARHL and adds to the rapidly growing literature on the cognitive consequences of ARHL.

## 1 Introduction

Age-related hearing loss (ARHL), or presbycusis, is a common condition involving gradual hearing loss due to aging (National Institute on Deafness and Other Communication, 2023). The increasing prevalence of ARHL globally is resulting in a major public health burden (Haile et al., 2021; World Health Organization, 2021). In the United States alone, a third of adults between 65 to 74 years and half over 75 years have ARHL (National Institute on Deafness and Other Communication, 2023). It is estimated that by 2030, ARHL will have an economic impact of 30 billion dollars (Stucky et al., 2010). Research in the past decade has shown that not only do individuals with ARHL experience typical auditory processing challenges, such as recognition of speech-in-noise (SiN), but they also experience non-auditory cognitive challenges beyond those seen with typical aging (CHABA, 1988; Lin, 2011; Humes, 2013; Powell et al., 2022; Shende and Mudar, 2023). Emerging work, including studies conducted by our group, has shown cognitive alterations even in those with mild ARHL in cognitive control assessed using visual modality (Shende et al., 2021, 2022).

Cognitive control refers to various mental operations that allow prioritization of information to accomplish target goals (Diamond, 2013; Mackie et al., 2013). An important cognitive control process is “value-directed strategic processing,” which refers to the preferential processing of salient information of higher value (Castel, 2007; Nguyen et al., 2019). This process is critical for efficient information processing in all sensory modalities. For instance, we strategically attend to warning signs related to hazardous conditions when driving on a freeway in a snowstorm compared to general information signs. In laboratory settings, value-directed strategic processing has been typically examined using visual word-list learning tasks (Castel, 2007; Castel et al., 2009; Murphy et al., 2024). In these tasks, words are paired with different point values ranging from low- to high-, such as 1–16 (1: low-value; 16: high-value), and participants are asked to recall as many words as they can with the goal of scoring maximum points. Typically, participants who perform well on these tasks recall more words of higher value, suggesting preferential value-based processing of information (Castel et al., 2007; Nguyen et al., 2019, 2020; Schwartz et al., 2023). It is important to note that the pairing of words with point values distinguishes strategic processing tasks from episodic learning tasks that use a list learning paradigm to measure recall irrespective of value as a measure of episodic learning (Castel et al., 2011; Elliott et al., 2020).

Value-directed strategic processing subsumes other attentional processes, including selective attention (Anderson, 2013). In the context of ARHL, selective attention has been long examined (Barr and Giambra, 1990; Craik, 2007; Shinn-Cunningham and Best, 2008; Van Gerven and Guerreiro, 2016; Dai et al., 2018), especially in the context of speech recognition (Humes and Dubno, 2010; Nuesse et al., 2018; Fuglsang et al., 2020; Shechter Shvartzman et al., 2022). On various tasks, such as coordinate response measure (Humes et al., 2006, 2017), attentive matrices (Bonmassar et al., 2022), *n-* back with distracting stimuli (Guerreiro and Van Gerven, 2017), Stroop (Guerreiro and Van Gerven, 2017; Gillingham et al., 2018; Ren et al., 2019; Huber et al., 2020; Chavant and Kapoula, 2022), and dichotic listening tasks (Shinn-Cunningham and Best, 2008; Torrente et al., 2022), studies have found alterations in selective attention in older adults with ARHL relative to younger and older adults with normal hearing. These findings suggest that changes in individuals with ARHL go beyond typical age-related declines in attention. Considering that these alterations have been found even on visual tasks, these findings suggest a more modality-general change in attentional processing in this population.

In contrast to the large evidence body on selective attention changes, value-directed strategic processing has remained largely unexplored in ARHL, with the exception of a study by our group (Shende et al., 2022). In our study, we examined behavioral changes in value-directed strategic processing in individuals with mild untreated ARHL relative to normal hearing (NH) controls with comparable age and education. We used an in-house developed value-directed list learning task where words were paired with binary values (10: high-value; 1: low-value) differentiated by letter case (Upper vs. Lower Case). See (Nguyen et al., 2019) for details of task development. Our results revealed that our untreated mild ARHL group recalled fewer high-value words relative to the NH controls, suggesting behavioral alterations in value-directed strategic processing beyond typical age-related cognitive control changes. Given that we used a visual task, these behavioral changes in individuals with ARHL offered preliminary evidence for modality-general alterations in value-directed strategic processing. Whether there are underlying neural alterations linked to these behavioral changes as one would expect based on theoretical postulations, such as information degradation (CHABA, 1988; Schneider and Pichora-Fuller, 2000), sensory deprivation (Lindenberger and Baltes, 1994; Baltes and Lindenberger, 1997), and common cause hypotheses (CHABA, 1988; Lindenberger and Baltes, 1994; Baltes and Lindenberger, 1997), remains unknown.

Event-related encephalography (EEG) is a useful technique to capture real-time temporal unfolding of value-directed strategic processing from the neurophysiological standpoint since it taps into neural activity time-locked to specific events (Sur and Sinha, 2009). Particularly measures linked to event-related spectral perturbations (ERSPs) capture both phase-locked and non-phase-locked spectral activity in the EEG signal (Makeig, 1993; Makeig et al., 2004). Using a value-directed strategic processing task, our group examined ERSPs in the theta and alpha bands in both cognitively healthy controls (Nguyen et al., 2019, 2020) and older adults with mild cognitive impairment (Nguyen et al., 2022). Overall, we found oscillatory activity in theta and alpha bands linked to strategic processing. Specifically, we found greater theta synchronization (more positive power relative to baseline) during processing of low-value relative to high-value words in frontal electrodes (Nguyen et al., 2019, 2020, 2022). Extant literature suggests that frontal theta activity is linked to cognitive control in general [see (Cavanagh and Frank, 2014) for a review] and is typically recorded at Fz and FPz electrodes [see (Mitchell et al., 2008)]. In particular, studies have linked frontal theta to general processing (Eschmann et al., 2020), working memory (Itthipuripat et al., 2013), and inhibitory control (Nigbur et al., 2011; Cavanagh and Frank, 2014), all of which are integral to value-directed strategic processing. We also found greater alpha desynchronization (more negative power relative to baseline) during processing of high- relative to low-value words in centroparietal and parietal electrodes in our study on value-directed strategic processing (Nguyen et al., 2019, 2020). Numerous studies have suggested that alpha desynchronization reflects information processing (Benedek et al., 2014; Fumuro et al., 2015) and selective attention (Klimesch et al., 1997, 2007).

In the context of ARHL, there is an emerging body of work on ERSPs (Petersen et al., 2015; Price et al., 2019) in relation to SiN recognition (Price et al., 2019) and auditory working memory tasks (Petersen et al., 2015). Findings suggest changes in alpha band power in those with ARHL relative to controls, specifically increased alpha power with mild and moderate degrees of hearing loss (Petersen et al., 2015). Studies have also found association between listening in challenging/noisy environments and parietal alpha power (Dimitrijevic et al., 2019; Wisniewski et al., 2021). In cochlear implant users, lower alpha desynchronization has been associated with increased listening effort (Dimitrijevic et al., 2019). In comparison to alpha oscillation, examination of theta in ARHL has received very little attention with some exceptions (Griffiths et al., 2020). To the best of our knowledge, none have examined theta and alpha oscillations linked to cognitively demanding tasks, such as value-directed strategic processing tasks, in this population.

The goal of the current study was to examine differences in theta and alpha power in individuals with unaided mild ARHL relative to NH controls of comparable age and education using our visual value-directed strategic processing task. Guided by the literature, including emerging work on neural oscillations in ARHL, and our previous studies on value-directed strategic attention in cognitively healthy and impaired populations, we hypothesized that the mild ARHL group would show lower theta synchronization and alpha desynchronization relative to the control group. To clarify, given the lack of studies on theta ERSPs in ARHL, our hypothesis of lower theta synchronization in the ARHL relative to the control group was guided by findings of our previous study (Nguyen et al., 2022), as well as expectation of poorer cognitive control in ARHL given that studies have shown poorer performance on cognitive control tasks in those with ARHL relative to NH controls (Lin, 2011; Powell et al., 2022; Shende and Mudar, 2023). We also hypothesized greater theta synchronization for low- versus high-value words and greater alpha desynchronization for high- versus low-value words. It is important to mention that we focused on individuals with unaided mild ARHL to understand whether mild alterations in hearing, which typically go untreated, impact neural activity linked to strategic processing. Furthermore, we intentionally chose to use a visual paradigm to minimize any confounds related to unaided hearing loss, and to ascertain whether neural changes, if observed, are modality-general in those with ARHL.

## 2 Materials and methods

### 2.1 Participants

19 older adults with untreated bilateral mild ARHL (11 female; mean age: 71.53 ± 8.04 years; mean education: 17.58 ± 3.53 years), and 17 NH controls (12 female; mean age: 67.76 ± 4.92 years; mean education: 18.06 ± 1.56 years) of comparable age and education participated in the study. All were right-handed and native English speakers without any history of communication disorders, learning disabilities, neurological disorders, traumatic brain injury, psychiatric disorders, or uncorrected visual impairment. Those with a history of substance abuse, use of psychoactive medications, other known etiologies of hearing loss such as noise-induced, injury-related, or ototoxicity, or other hearing-related issues such as unilateral and/or bilateral continuous tinnitus, conductive or mixed hearing loss, and those with hearing aid use were excluded. All participants signed a written informed consent in accordance with protocols approved by the Institutional Review Board of the University of Illinois Urbana-Champaign (protocol # 17067) before completing the study. Demographic information for both groups is reported in Table 1.

**Table 1:** Participant demographics.

	ARHL	NH	*p*
N	19	17	–
Age (years)	71.53 (8.04)	67.76 (4.92)	0.105
Education (years)	17.58 (3.53)	18.06 (1.56)	0.609
Sex	11F/8 M	12F/5 M	0.330

Cells represent mean (standard deviation) except N and Sex which represent the sample size and sex distribution, respectively. ARHL, age-related hearing loss; NH, normal hearing. ^*^p ≤ 0.05.

### 2.2 Tasks and procedures

#### 2.2.1 Baseline

All participants were asked if they had any vision-related difficulties. Those who reported issues despite having corrected vision were excluded since we used a visual task in this study to examine value-directed strategic processing. Additionally, all study participants underwent cognitive and mental health screenings. A global cognitive screener, the Montreal Cognitive Assessment Scale (MoCA) (Nasreddine et al., 2005), was used for cognitive screenings (MoCA scores, ARHL group: 26.95 [2.09]; NH group: 28.24 [1.20]), and the Geriatric Depression Scale (GDS) (Almeida and Almeida, 1999) was used to conduct the mental health screenings (GDS scores, ARHL group: 0.78 [1.03]; NH group: 0.29 [0.58]).

#### 2.2.2 Audiological evaluation

A comprehensive audiological examination was conducted for all participants. Otoscopic evaluation was conducted to visually inspect for external ear and tympanic membrane pathologies/disorders; participants with these issues were excluded. Tympanometry and acoustic reflexes were examined to ensure normal middle ear function and to rule out reflex pathway abnormalities. Pure-tone audiometry was performed to obtain hearing thresholds, with air conduction thresholds determined from 0.25 to 8 kHz in decibel hearing level (dB HL) using the modified Hughson-Westlake method (Carhart and Jerger, 1959). Insert earphones were used as transducers with the Equinox 2.0 audiometer, which was calibrated to the American National Standards Institute S3.6 2010 standards. We calculated pure-tone average (PTA) using an average of air conduction thresholds at 0.5, 1, 2, and 4 kHz in each ear and operationally defined NH as ≤25 dB HL PTA in the better ear and hearing loss as >25 dB HL PTA in the better ear (Lin, 2011).

**Figure 1:**
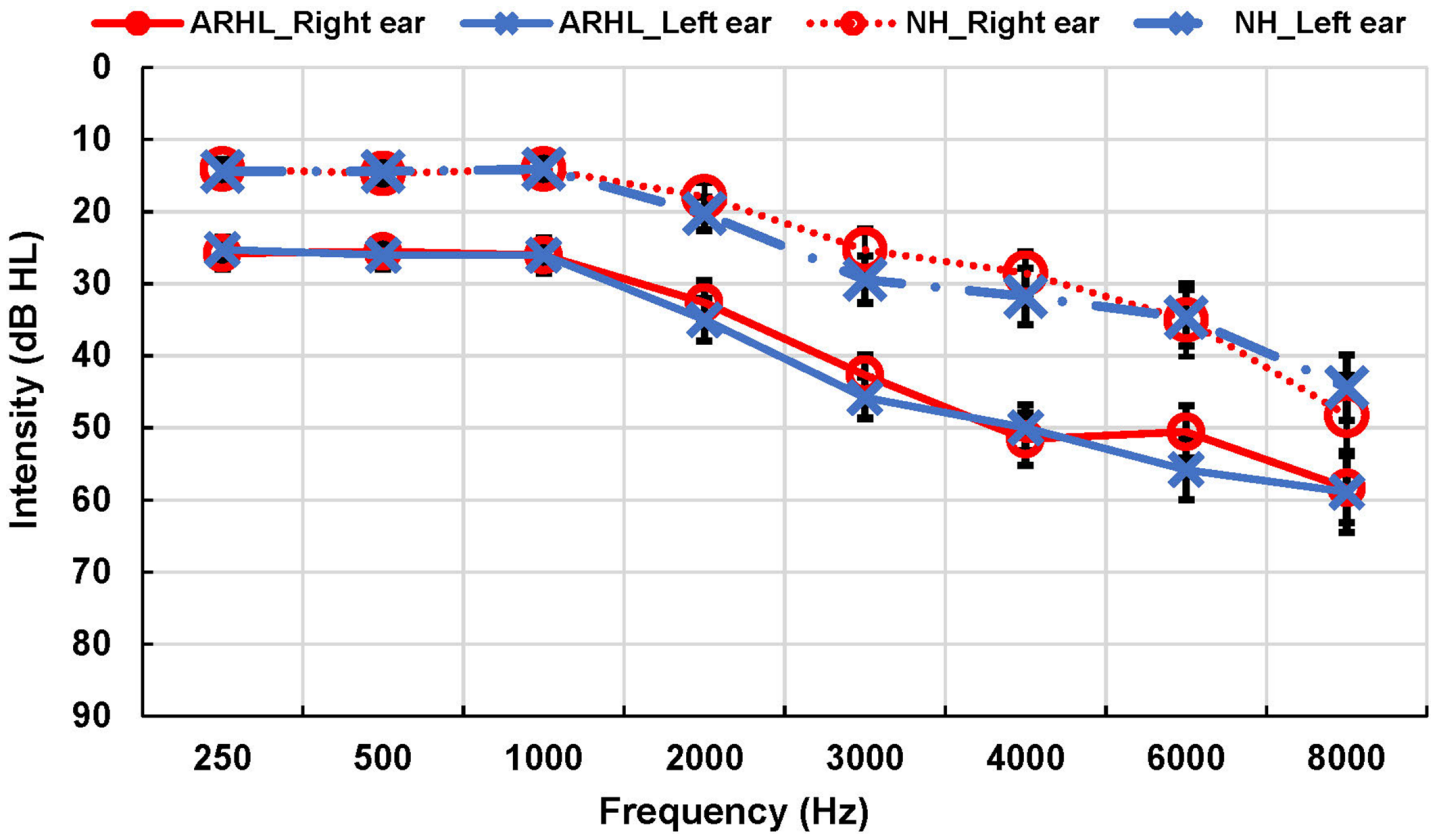
Audiograms for ARHL and NH groups. Average of air conduction hearing thresholds for both ears across frequencies in ARHL and NH groups. Error bars represent standard errors. ARHL, age-related hearing loss; NH, normal hearing.

Speech audiometry was conducted to determine the participant’s acuity to speech sounds. Evaluation included obtaining Speech Reception Thresholds (SRTs) using spondee words and Word Recognition Score (WRS) using Northwestern University list 6 (NU-6) (Tillman and Carhart, 1966) in each ear. Additionally, we examined participants’ ability to recognize SiN using the Quick Speech-in-Noise test (QuickSIN) (Killion et al., 2004) in each ear separately (monoaural condition) and both ears simultaneously (binaural condition). The task required participants to repeat back sentences presented against multi-talker babble at signal-to-noise ratios (SNRs) that varied from +25 dB to 0 dB in 5 dB steps. QuickSiN scores of SNR loss were recorded, with higher scores suggesting worse SiN recognition. Most comfortable and uncomfortable listening levels were obtained to ensure the audibility of speech stimuli for each participant. QuickSiN sentences were presented at 70 dB HL as recommended or at the participant’s most comfortable level if 70 dB HL presentation level was uncomfortable. All audiometric testing was performed in a sound-treated booth in a two-room setup. Group means for all audiological measures are reported in Table 2. Audiograms for NH and ARHL are shown in Figure 1.

**Table 2:** Group means for audiological measures.

	ARHL	NH	*p*
Better ear PTA	32.63 (7.05)	17.13 (5.43)	<0.001^*^
Right SRT (dB HL)	31.05 (9.8)	20.29 (5.44)	<0.001^*^
Left SRT (dB HL)	31.58 (6.88)	19.12 (5.92)	<0.001^*^
Right WRS (%)	94.11 (11.10)	98.82 (2.74)	0.098
Left WRS (%)	94.11 (8.81)	98.59 (4.88)	0.074
Right QuickSIN	6.05 (3.28)	2.73 (1.85)	<0.001^*^
Left QuickSIN	5.5 (3.32)	3.05 (2.06)	0.013^*^
Binaural QuickSIN	3.60 (3.01)	1.58 (1.12)	0.014^*^

Cells represent means of raw test scores (standard deviation). ARHL, age-related hearing loss; NH, normal hearing; PTA (dB HL), pure-tone average (decibels hearing level); SRT (dB HL), speech reception threshold (decibels hearing level); WRS (dB HL), word recognition score (decibels hearing level); QuickSIN, quick speech-in-noise.^*^p ≤ 0.05.

#### 2.2.3 Strategic processing

Following the audiological evaluation, participants completed a value-directed strategic processing task, which is a variation of a word list learning task (Nguyen et al., 2019, 2020). Stimuli consisted of 200 single-syllable four-letter nouns controlled for imageability, concreteness, frequency, and familiarity, divided into five lists of 40 words each, with each list consisting of a different set of words. In each list, half of the words (*n* = 20) were assigned to the “high-value” condition (worth 10 points), and half (*n* = 20) were assigned to the “low-value” condition (worth 1 point). To differentiate the value of the words, letter case was used, wherein all letters of the word were written in either lowercase (e.g., shoe) or uppercase (e.g., SHOE) letters. The font size was controlled so that lowercase and uppercase letters all appeared the same size on the computer screen. The word order was pseudorandomized for each list. See (Nguyen et al., 2019) for additional details related to the task. We developed four versions of the task, which were counterbalanced for word value and letter case, such that two versions had high-value words presented in lowercase letters and low-value words presented in uppercase letters, and two versions had high-value words presented in uppercase letters and low-value words presented in lowercase letters. Random assignments of the versions were done across participants.

Participants saw the following instructions on a computer screen: “You will see words appear on the screen one at a time. Some words are in uppercase, and some words are in lowercase. The uppercase words *[or lowercase words]* are worth 10 points each (high-value words). The lowercase words *[or uppercase words]* are worth 1 point each (low-value words). At the end of the list you will see the word “REMEMBER” on the screen. Your task is to remember as many of the words from the list as possible with the goal of scoring the maximum number of points. This is similar to a game in which words are worth different amounts of money.” Task understanding was ensured, including the point values for the uppercase and lowercase words depending on the assigned version. It is critical to note that no specific instructions were provided to the participants on how to be strategic, for instance, by only focusing on the high-value words.

**Figure 2:**
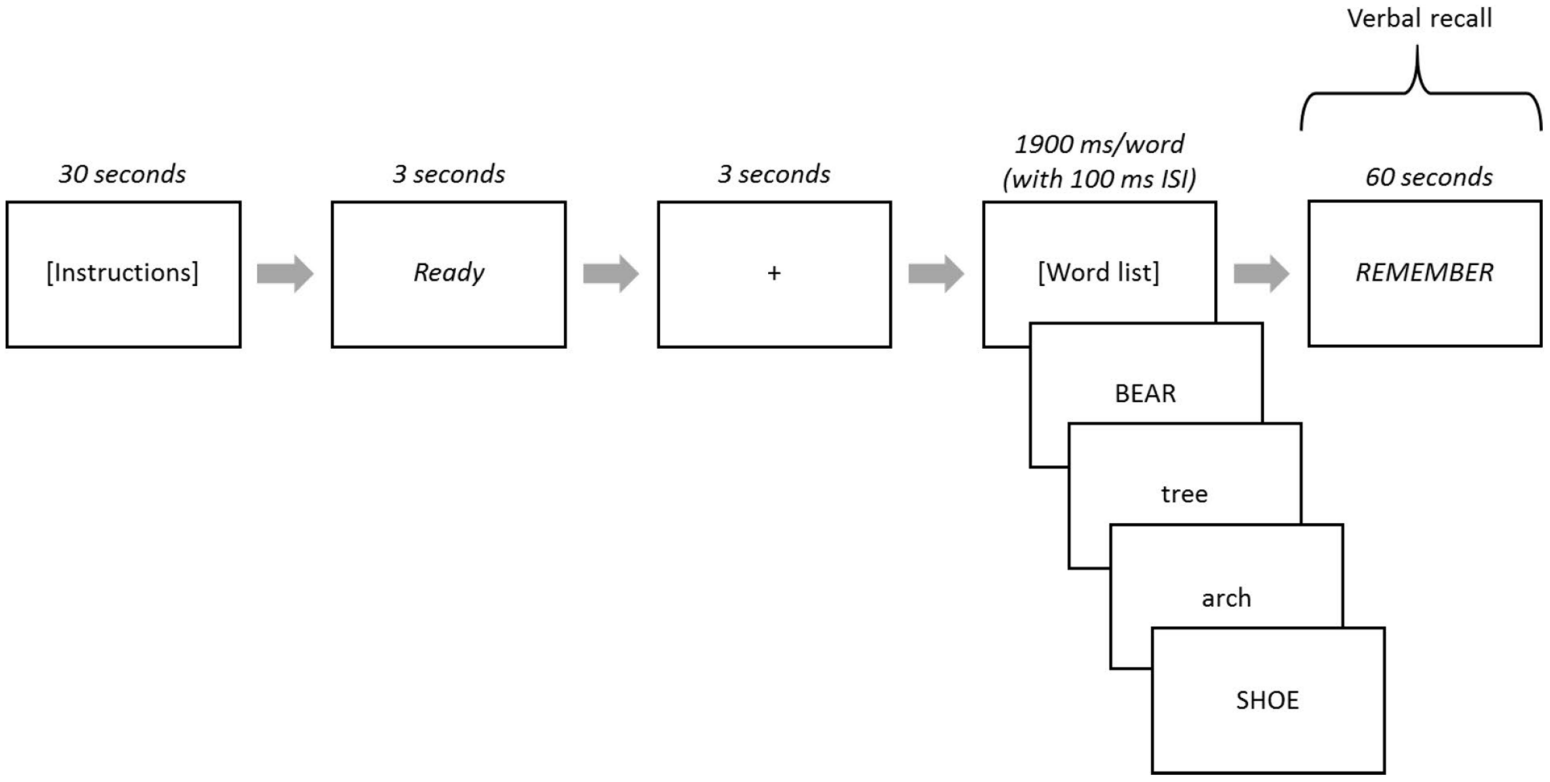
Strategic processing task schematic. Lowercase or uppercase words served as high- or low-value words depending on task version. When the word “REMEMBER” was presented, participants verbally recalled words from that list. Responses were recorded on paper and scored for each of the five lists.

After the task instructions, the word “Ready” appeared in the center of the computer screen for 3 s, subsequent to which a fixation (+) was presented for 3 s. Then, the 40 words (in each list) were displayed one-at-a-time in the center of the computer screen for a duration of 1900 ms each with an inter-stimulus interval of 100 ms (blank screen). The word “REMEMBER” appeared at the end of each list and remained on the screen for 60 s, during which participants recalled as many words as they could, with their responses recorded on a score sheet by the experimenter (see Figure 2 for task schematic). Each participant received immediate feedback about their recall score after each list and before the presentation of the next list.

#### 2.2.4 EEG data collection and pre-processing

Continuous EEG was recorded while participants performed the strategic processing task, using a 64-electrode elastic cap (Neuroscan Quikcap) with the Neuroscan SynAmpsRT amplifier and Scan v4.5 software (sampling rate: 1 kHz, bandpass filter: DC-200 Hz). Impedances for all electrodes were kept below 10kΩ. The reference electrode was located in a midline position between Cz and CPz, and the vertical electrooculogram was recorded at sites below and above the left eye. Raw EEG data from all five word lists (obtained during a single recording session) were appended together to have 100 trials per condition (i.e., high-value and low-value words), and data were processed offline. Poorly functioning electrodes were identified by high impedance values as well as visual inspection and were excluded from analyses (average number of electrodes excluded: 0.73 in ARHL group, 1.23 in the NH group; no group differences noted, *F* [1,34] = 3.33, *p* = 0.077). Eye blinks were corrected using spatial filtering in Neuroscan. The data were epoched from 500 ms before stimulus onset to 1,500 ms after stimulus offset. Epochs with peak signal amplitudes of ±75 μV were rejected (rejection rates: 17.84% for high-value in ARHL group, 16.58% in NH group, *F* [1,34] = 0.094, *p* = 0.761; 18.26% for low-value in ARHL group, 16.58% in NH group, *F* [1,34] = 0.094, *p* = 0.761). EEG data were then re-referenced to the average potential over the entire scalp.

#### 2.2.5 ERSP analysis

ERSPs were analyzed from 0 to 1,000 ms (post-stimulus onset) using a non-overlapping baseline of −400 to −100 ms (pre-stimulus onset). EEGLAB toolbox (Version 14.1.1b) (Delorme and Makeig, 2004) running under Matlab 2016b (MathWorks, Natick, MA, United States) was used to analyze the data. Time-frequency decomposition was conducted using short-time Fourier transform with Hanning window tapering as implemented in the EEGLAB function *newtimef.m* (Delorme and Makeig, 2004). Time-frequency data were obtained using a 256-ms sliding window with a step-size of 10 ms and a pad ratio of 4 resulting in a frequency resolution of approximately 1 Hz. Baseline correction was done in accordance with a gain model (Delorme and Makeig, 2004; Grandchamp and Delorme, 2011) where each time-frequency time point was divided by the average pre-stimulus baseline power from −400 to −100 ms relative to stimulus onset at the same frequency.

#### 2.2.6 ERSP power estimation

**Figure 3:**
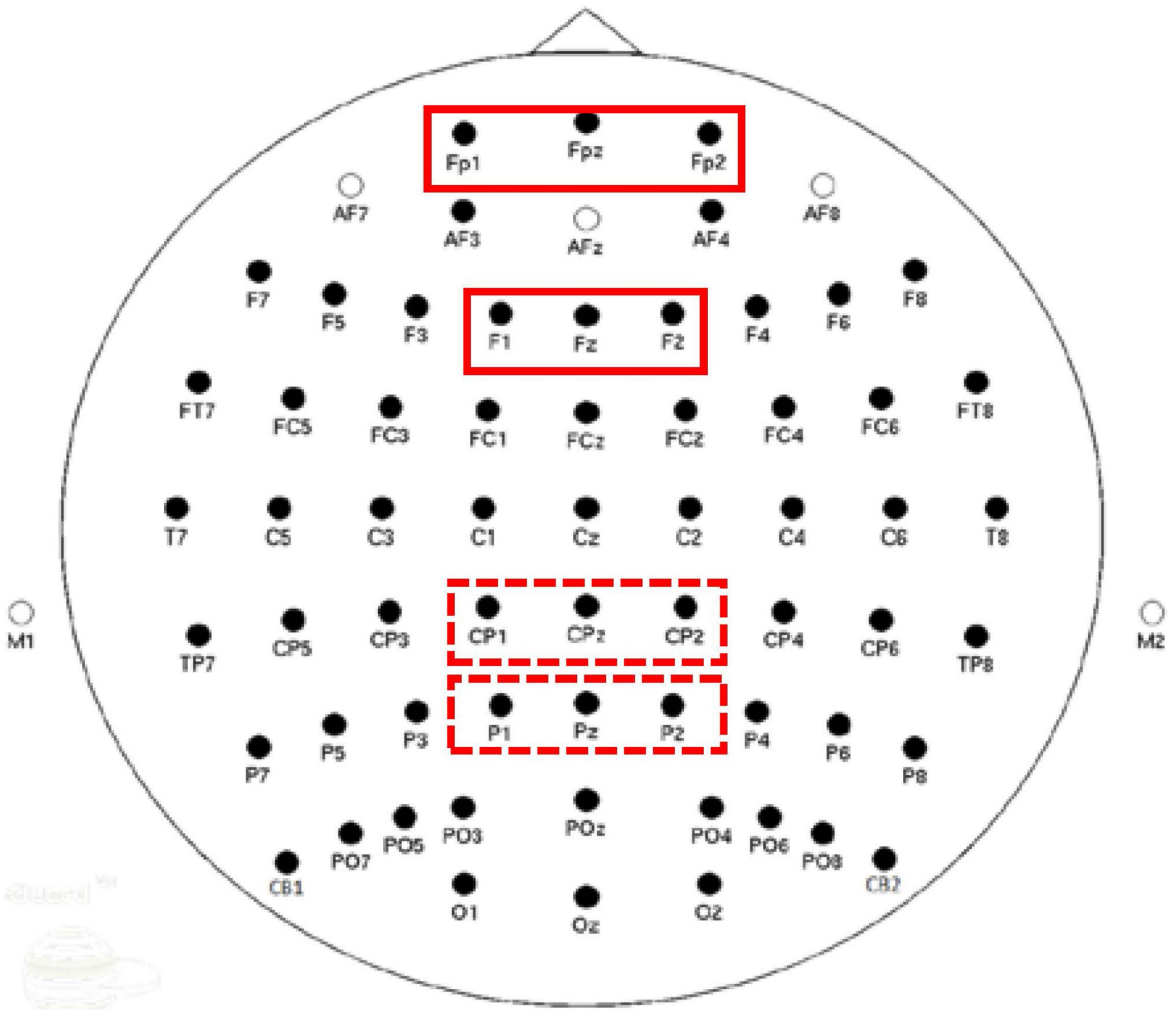
Electrode clusters used for Event-Related Spectral Perturbations (ERSP) power estimation. Solid boxes represent electrode clusters for theta analyses and dotted boxes represent clusters for alpha analyses.

We estimated mean power in the theta band (4–8 Hz) at prefrontal (FP1, FPz, FP2) and frontal (F1, Fz, F2) electrode clusters; and in the alpha band (8–12 Hz) at centroparietal (CP1, CPz, CP2) and parietal (P1, Pz, P2) electrode clusters. Figure 3 illustrates electrode clusters used for analysis. The selection of these electrode sites was guided by our previous work, as well as the literature, which shows greater prominence of theta band at frontal sites and alpha band at parietal sites (Pfurtscheller and Lopes da Silva, 1999; Kawasaki et al., 2010; Cavanagh and Frank, 2014; Nguyen et al., 2019, 2020, 2022). Given that this is the first study examining ERSP correlates of value-directed strategic processing in ARHL, we decided to explore additional electrode sites, specifically prefrontal and centroparietal electrodes (Mitchell et al., 2008; Nguyen et al., 2022). Change in power will be described as synchronization or desynchronization, depending on an increase or decrease in the power, respectively, relative to baseline. Mean power was computed for high- and low-value conditions in 100 ms time windows from 0 ms to 1,000 ms, resulting in 10 time windows for analysis.

### 2.3 Statistical analysis

All data were analyzed using IBM SPSS Statistics (Version 26). For the audiological measures, General Linear Models (GLMs) were used, with group (ARHL/NH) as a between-subject variable and the audiological measures as within-subject variables. For the strategic processing task, behavioral data combined across all five lists (total number of words recalled) were examined using a GLM, with group (ARHL/NH) as a between-subject factor and value (high−/low-) as a within-subject factor. We also examined group differences in total points earned.

ERSP data were examined using separate GLMs for theta and alpha mean power for each of the 10 time windows (100 ms time windows between 0 and 1,000 ms post-stimulus). For this, group (ARHL/NH) was used as a between-subject factor, and value (high−/low-) as a within-subject factor. The Bonferroni method was used to correct for multiple comparisons with a threshold of *p* ≤ 0.05. The reported *p*-values, where not specified otherwise, are derived from *F*- and *t-* statistics.

## 3 Results

### 3.1 Task-related behavioral data

**Figure 4:**
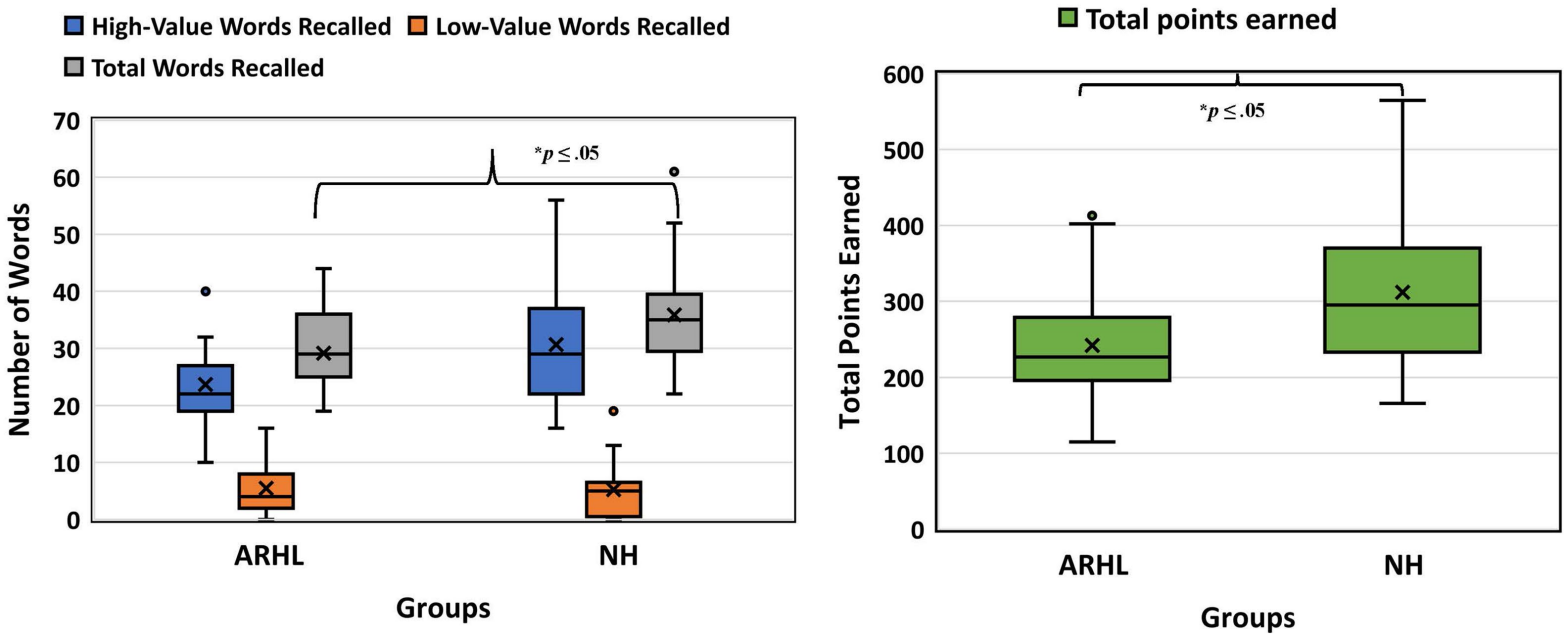
Boxplots depicting behavioral data. The figure on the left depicts boxplots for number of words recalled and figure on the right depicts boxplots for total points earned. Horizontal lines represent median power, with boxes representing the interquartile range and whiskers extending to minimum and maximum values. ARHL, age-related hearing loss, NH, normal hearing. ^*^p ≤ 0.05.

Behavioral data showed a main effect of group for the total number of words recalled, *F*(1,34) = 5.639, *p* = 0.023, with the ARHL group recalling fewer total words than the NH group (total # words recalled: ARHL group = 29.15 [7.15]; NH group = 35.88 [9.76]). A significant main effect of value was also observed, *F*(1,34) = 117.288, *p* < 0.001, with more high-value words recalled (ARHL group = 23.68 [7.79]; NH group = 30.65 [10.71]) than low-value words (ARHL group = 5.47 [4.53]; NH group = 5.24 [4.96]). No other effects were significant when examining the total number of words. Significant group differences were observed for total points recalled, *F*(1,34) = 5.219, *p* = 0.029, with the ARHL group scoring lower total points than the NH group (ARHL group = 242.31 [76.13]; NH group = 311.70 [105.21]). See Figure 4.

### 3.2 Theta band (4–8 Hz) mean power

**Figure 5:**
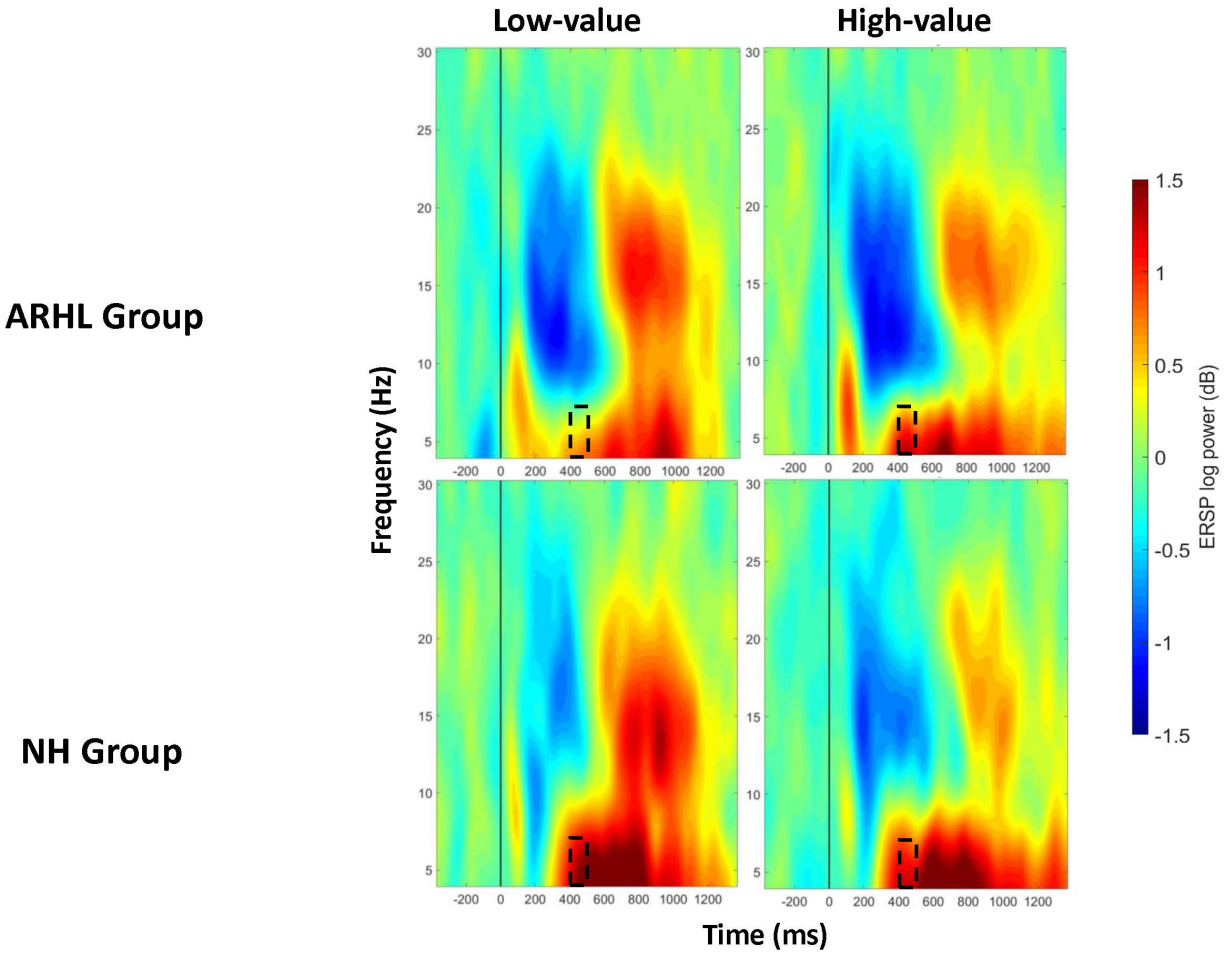
ERSP comparisons for theta band at prefrontal electrode cluster for interaction effects between group and value. Spectrograms illustrate differences between groups (ARHL/NH) and value (high-low-value) for theta band (4 to 8 Hz) at the prefrontal electrode cluster. The 0 ms time point (solid vertical line) represents stimulus onset. Dashed black rectangles indicate the time windows in which significant interaction effects between group and value were observed (also see Table 5). ARHL, age- related hearing loss; NH, normal hearing.

ERSP data showed a main effect of group between 100 and 200 ms post-stimulus onset in the prefrontal electrode cluster, with greater theta synchronization in the ARHL relative to NH group (*p* = 0.046; Table 3; Supplementary Figure S1). A significant main effect of value was observed at the frontal electrode cluster, between 200–300 ms, and 700–1000 ms post-stimulus onset (*p* < 0.05), with greater theta synchronization for low- than high-value words (Table 4; Supplementary Figure S2). Significant interaction effects between group and value were observed between 400 and 500 ms post-stimulus onset in the prefrontal electrode cluster (*p* = 0.011; Table 5; Figure 5). Also see Supplementary Figure S3 for the depiction of individual variability for this significant group by value interaction effect. While the post-hoc analyses did not reveal any significant between-group differences (*p* > 0.05), within-group differences between high- and low-value words were observed in the ARHL group (*p* = 0.019) with greater theta synchronization for high- compared to low-value words. No such differences were observed within the NH group. Results for ERSP data, including theta findings, are reported in Tables 3–5.

**Table 3:** Statistical results for main effects of group for theta and alpha band mean power.

	Time (ms)									
	0–100	100–200	200–300	300–400	400–500	500–600	600–700	700–800	800–900	900–1,000
Theta										
*Prefrontal*	*F* = 2.237	*F* = 4.283	*F* = 0.429	*F* = 1.759	*F* = 1.920	*F* = 1.972	*F* = 1.101	*F* = 1.279	*F* = 0.268	*F* = 0.039
	*p* = 0.144	*p* = 0.046^*^	*p* = 0.517	*p* = 0.194	*p* = 0.175	*p* = 0.169	*p* = 0.302	*p* = 0.266	*p* = 0.608	*p* = 0.844
*Frontal*	*F* = 0.391	*F* = 0.030	*F* = 0.026	*F* = 1.392	*F* = 1.061	*F* = 0.933	*F* = 0.639	*F* = 0.260	*F* = 0.351	*F* = 1.726
	*p* = 0.536	*p* = 0.864	*p* = 0.874	*p* = 0.246	*p* = 0.310	*p* = 0.341	*p* = 0.430	*p* = 0.614	*p* = 0.557	*p* = 0.198
Alpha										
*Centro-parietal*	*F* = 2.941	*F* = 0.233	*F* = 0.041	*F* = 0.096	*F* = 0.035	*F* = 0.061	*F* = 0.090	*F* = 0.011	*F* = 0.115	*F* = 0.235
	*p* = 0.095	*p* = 0.633	*p* = 0.841	*p* = 0.759	*p* = 0.852	*p* = 0.806	*p* = 0.765	*p* = 0.918	*p* = 0.736	*p* = 0.631
*Parietal*	*F* = 0.068	*F* = 0.021	*F* = 0.002	*F* = 0.502	*F* = 0.204	*F* = 0.367	*F* = 0.025	*F* = 0.071	*F* = 0.091	*F* = 0.013
	*p* = 0.795	*p* = 0.886	*p* = 0.962	*p* = 0.484	*p* = 0.654	*p* = 0.549	*p* = 0.876	*p* = 0.791	*p* = 0.765	*p* = 0.911

Cells display statistics for the main effects of group (ARHL/NH) for mean power in theta (4–8 Hz) and alpha (8–12 Hz) bands across 10 time windows post-stimulus onset. For all F-values, degrees of freedom = (1, 34). Significant main effects of value are indicated by highlighted cells. ARHL, age-related hearing loss; NH, normal hearing.^*^p ≤ 0.05.

**Table 4:** Statistical results for main effects of value for theta and alpha band mean power.

	Time (ms)									
	0–100	100–200	200–300	300–400	400–500	500–600	600–700	700–800	800–900	900–1,000
Theta										
*Prefrontal*	*F* = 0.416	*F* = 0.556	*F* = 0.606	*F* = 0.773	*F* = 0.687	*F* = 0.015	*F* = 0.010	*F* = 1.073	*F* = 0.326	*F* = 1.959
	*p* = 0.523	*p* = 0.461	*p* = 0.442	*p* = 0.385	*p* = 0.413	*p* = 0.902	*p* = 0.921	*p* = 0.308	*p* = 0.572	*p* = 0.171
*Frontal*	*F* = 0.000	*F* = 0.042	*F* = 7.295	*F* = 0.451	*F* = 0.972	*F* = 0.850	*F* = 2.055	*F* = 17.989	*F* = 5.679	*F* = 6.263
	*p* = 0.984	*p* = 0.839	*p* = 0.011*	*p* = 0.507	*p* = 0.331	*p* = 0.363	*p* = 0.161	*p* = 0.000*	*p* = 0.023*	*p* = 0.017*
Alpha										
*Centro-parietal*	*F* = 1.294	*F* = 0.055	*F* = 0.632	*F* = 3.580	*F* = 9.167	*F* = 8.811	*F* = 17.75	*F* = 48.169	*F* = 13.667	*F* = 4.671
	*p* = 0.263	*p* = 0.816	*p* = 0.432	*p* = 0.067	*p* = 0.005*	*p* = 0.005*	*p* = 0.000*	*p* = 0.000*	*p* = 0.001*	*p* = 0.038*
*Parietal*	*F* = 0.045	*F* = 1.105	*F* = 4.287	*F* = 3.552	*F* = 0.922	*F* = 3.096	*F* = 14.843	*F* = 38.902	*F* = 16.963	*F* = 4.163
	*p* = 0.834	*p* = 0.301	*p* = 0.046*	*p* = 0.068	*p* = 0.344	*p* = 0.087	*p* = 0.000*	*p* = 0.000*	*p* = 0.000*	*p* = 0.050*

Cells display statistics for the main effects of value (high−/low-value words) for mean power in theta (4–8 Hz) and alpha (8–12 Hz) bands across 10 time windows post-stimulus onset. For all F-values, degrees of freedom = (1, 34). Significant main effects of value are indicated by highlighted cells.^*^p ≤ 0.05.

**Table 5:** Statistical results for the group by value interactions for theta and alpha band mean power.

	Time (ms)									
	0–100	100–200	200–300	300–400	400–500	500–600	600–700	700–800	800–900	900–1,000
Theta										
*Prefrontal*	*F* = 0.513	*F* = 0.078	*F* = 1.330	*F* = 0.006	*F* = 7.237	*F* = 2.679	*F* = 0.648	*F* = 0.054	*F* = 0.152	*F* = 0.284
	*p* = 0.479	*p* = 0.781	*p* = 0.257	*p* = 0.937	*p* = 0.011*	*p* = 0.111	*p* = 0.426	*p* = 0.818	*p* = 0.699	*p* = 0.597
*Frontal*	*F* = 0.670	*F* = 0.763	*F* = 2.025	*F* = 0.024	*F* = 1.692	*F* = 1.463	*F* = 0.104	*F* = 0.000	*F* = 0.164	*F* = 0.403
	*p* = 0.419	*p* = 0.388	*p* = 0.164	*p* = 0.878	*p* = 0.202	*p* = 0.235	*p* = 0.749	*p* = 0.985	*p* = 0.688	*p* = 0.530
Alpha										
*Centro-parietal*	*F* = 0.022	*F* = 0.329	*F* = 0.000	*F* = 0.233	*F* = 0.110	*F* = 0.974	*F* = 2.904	*F* = 4.582	*F* = 0.153	*F* = 0.262
	*p* = 0.884	*p* = 0.570	*p* = 0.990	*p* = 0.632	*p* = 0.742	*p* = 0.331	*p* = 0.098	*p* = 0.040*	*p* = 0.698	*p* = 0.612
*Parietal*	*F* = 0.763	*F* = 0.002	*F* = 0.008	*F* = 0.123	*F* = 0.409	*F* = 0.002	*F* = 0.158	*F* = 2.803	*F* = 0.199	*F* = 0.092
	*p* = 0.389	*p* = 0.962	*p* = 0.931	*p* = 0.728	*p* = 0.527	*p* = 0.965	*p* = 0.693	*p* = 0.103	*p* = 0.658	*p* = 0.764

Cells display statistics for the interaction effects between group (ARHL/NH) and value (high−/low-value words) for mean power in theta (4–8 Hz) and alpha (8–12 Hz) bands across 10 time windows post-stimulus onset. For all F-values, degrees of freedom = (1, 34). Significant main effects of value are indicated by highlighted cells. ARHL, age-related hearing loss, NH, normal hearing.^*^p ≤ 0.05.

### 3.3 Alpha band (8–12 Hz) mean power

**Figure 6:**
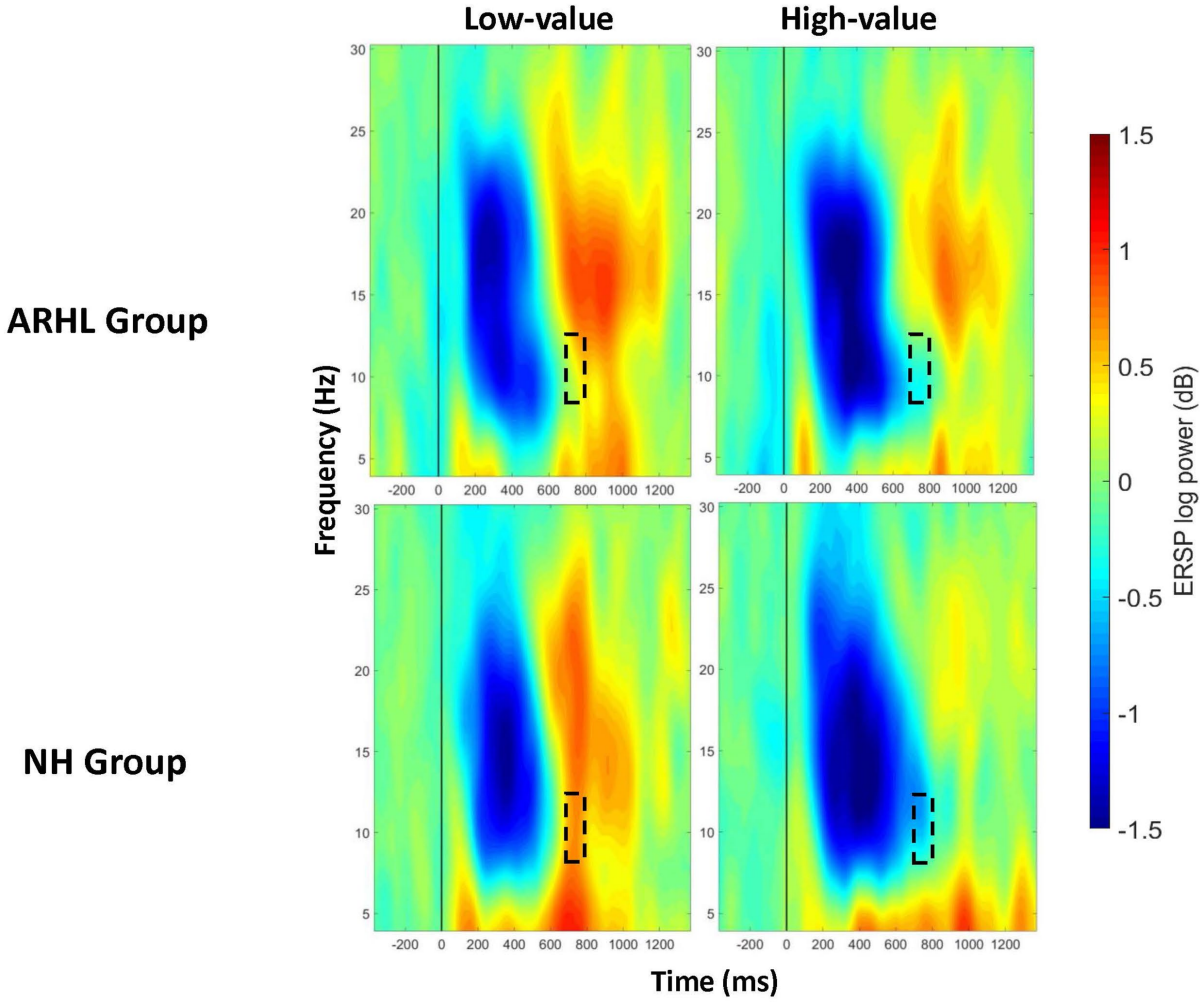
ERSP comparisons for alpha band at centroparietal electrode cluster for interaction effects between group and value. Spectrograms illustrate differences between groups (ARHL/NH) and value (high−/low-value) for alpha band (8–12 Hz) at the centroparietal electrode cluster. The 0 ms time point (solid vertical line) represents stimulus onset. Dashed black rectangles indicate the time windows in which significant interaction effects between group and value were observed (also see Table 5). ARHL, age-related hearing loss; NH, normal hearing.

**Figure 7:**
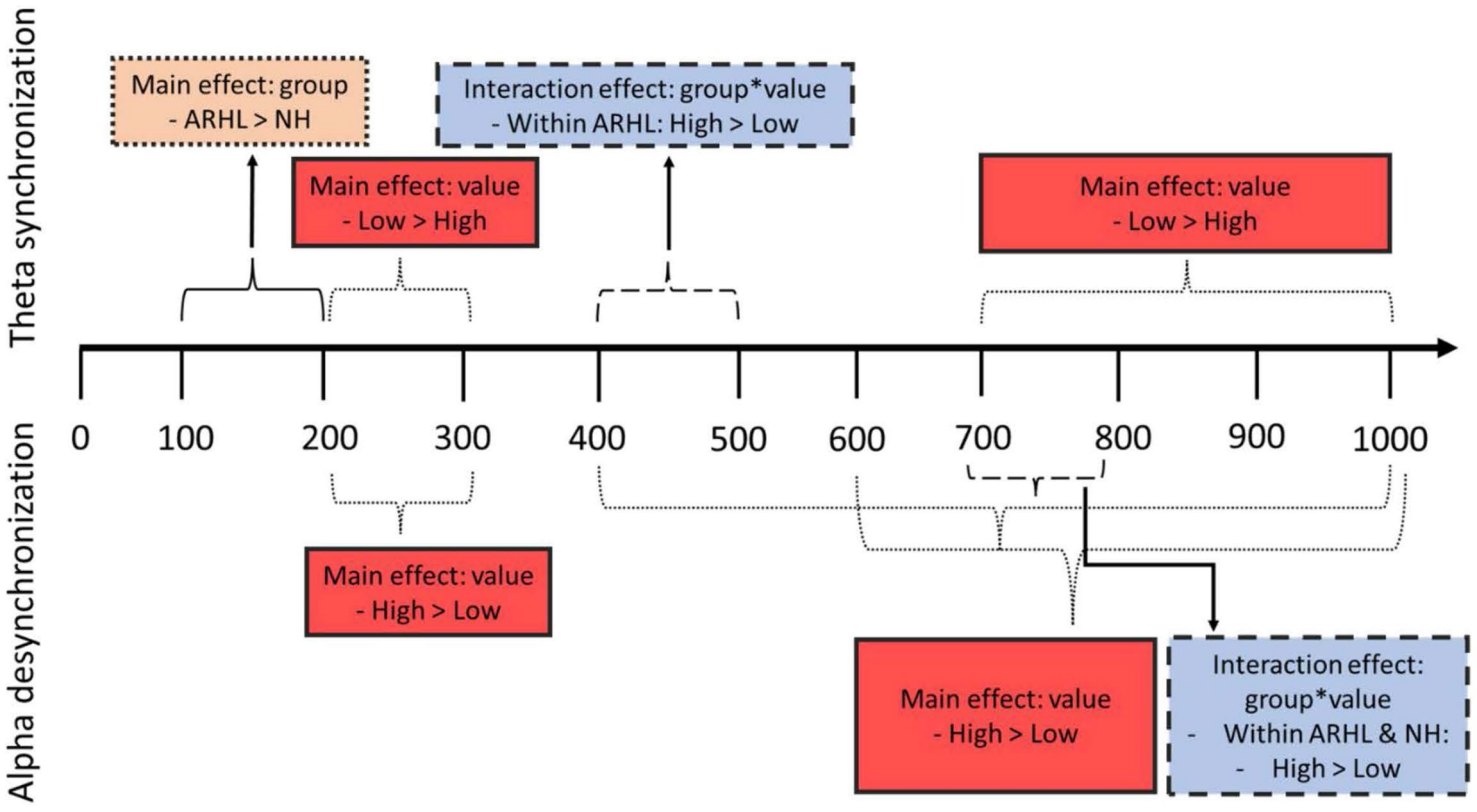
Schematic of main findings. Top part of the figure represents theta findings; bottom part shows alpha findings. Orange dotted box shows findings of main effect of group; red solid boxes show findings of main effect of value; blue dashed boxes show findings of interaction effect between group and value.

A significant main effect of value was observed between 400 and 1,000 ms at the centroparietal electrode cluster (Supplementary Figure S4), and between 200–300 ms and 600–1,000 ms at the parietal cluster, with greater alpha desynchronization for the high- compared to low-value words (Table 4; Supplementary Figure S5). A significant interaction between group and value was observed between 700 and 800 ms post-stimulus onset at the centroparietal electrode cluster (*p* = 0.040; Table 5; Figure 6; Also see Supplementary Figure S6 for the depiction of individual variability for this significant group by value interaction effect). Post-hoc analyses did not reveal any significant differences between groups, but there were significant within-group differences between high- and low-value words. These differences were seen in both ARHL and NH groups, with greater alpha desynchronization observed for high- compared to low-value words (*p* < 0.001). Alpha findings are reported in Tables 3–5. All significant ERSP findings in the time course are depicted in Figure 7.

## 4 Discussion

The current study examined ERSPs corresponding to a visual value-directed strategic processing task in older adults with ARHL relative to NH controls of similar age and education. Behaviorally, the ARHL group recalled fewer total words across all lists combined than the NH group. On EEG measures, two major findings related to group differences emerged: (1) the ARHL group had greater theta synchronization (100–200 ms) relative to the NH group during early processing periods; and (2) furthermore, the ARHL group had differences in theta synchronization (400–500 ms) for high- compared to low-value words, unlike the NH group. There were also some similarities observed in the two groups. Both groups showed greater alpha desynchronization for high- compared to low-value words (700–800 ms) although the magnitude of these differences was slightly more in the NH group. Also, both groups showed value-based differences in theta (200–300 ms; 700–1,000 ms) and alpha (600–1,000 ms).

Task-related behavioral data combined across all lists showed that the mild ARHL group recalled significantly fewer total number of words relative to the NH controls. These results could be attributed to just differences in episodic list learning between the groups. A few others have also found poorer episodic memory/list learning in individuals with ARHL relative to normal hearing controls, including on visual tasks of episodic memory (Rönnberg et al., 2011; Brewster and Rutherford, 2021; Tarawneh et al., 2022). Studies have shown that in ARHL, the brain undergoes functional reorganization (Campbell and Sharma, 2013; Campbell and Sharma, 2014; Lin et al., 2014; Armstrong et al., 2019), which can negatively affect the ability to retain information in memory (Loughrey et al., 2019). This is known as maladaptive plasticity, which has been found even in individuals with milder ARHL (Campbell and Sharma, 2014; Sharma and Glick, 2016; Slade et al., 2020). Changes in strategic processing likely contributed to the difficulties in list learning in individuals with ARHL relative to controls. Significant group difference in total points earned, with the ARHL group scoring lower total points than the NH controls, supports this speculation. To encourage strategic processing and learning of high-value words, participants were instructed to remember as many words as possible at the end of each list, with the goal of scoring a maximum number of points. However, participants were not given explicit instructions to focus on only high-value words since we wanted to examine inherent strategic processing ability. Our findings suggest that older adults with mild ARHL have compromised strategic processing compared to NH controls when point-values earned is used as a metric.

Task-related ERSP data revealed differences in neural processing between ARHL and NH groups in early time periods. Contrary to our hypothesis, we observed greater theta synchronization between 100 and 200 ms post-stimulus onset in the prefrontal electrode cluster in the ARHL group compared to NH controls. Theta synchronization in frontal regions has been linked to proactive and reactive control (Cooper et al., 2015; Messel et al., 2021; Pscherer et al., 2023). Proactive control allows one to adapt thoughts and behavior in anticipation of task goals, including any interference, whereas reactive control is a correction mechanism that involves detection and resolution of interference after its onset (Braver, 2012; Stuphorn and Emeric, 2012; Botvinick and Braver, 2015). Perhaps higher theta synchronization in the ARHL group relative to controls was related to the engagement of additional neural resources to support proactive control of task performance. This compensatory mechanism in the ARHL group to better support overall processing related to the task was still not sufficient to match their behavioral performance to NH controls, since they recalled fewer total number of words and earned lower total points. We have to be cautious in linking our ERSP findings to behavioral data given that behavioral data in our value-directed list learning paradigm includes cognitive processes beyond what is linked to strategic processing captured by the ERSP findings. In general, word recall on list learning tasks involves initial processing, learning/encoding, and subsequent retrieval. While our instructions promoted strategic processing, when EEG data were collected, participants were not asked to provide any overt response after each word in the list was presented. The task was designed this way intentionally to examine real-time strategic processing with minimal confounds from other cognitive processes (e.g., retrieval) or motoric response from a button press. We elicited recall at the end of each list to demonstrate that the behavioral results are consistent with value-directed strategic processing and learning behavioral literature (more high-value words recalled compared to low-value words). While behavioral data in terms of recall at the end of the list provides some insight into strategic processing, given that such recall involves cognitive processes related to word encoding and retrieval, not captured by the ERSPs, these findings need further validation. Future studies should consider how this task could be modified to collect ERSP data that can reveal possible associations between top-down cognitive processes of strategic processing and subsequent behavioral recall of high and low-value words to verify these findings. Higher theta synchronization in the ARHL relative to the control group between 100–200 ms could also point toward alterations in bottom-up sensory processing. Studies examining electrophysiological activity involving visual tasks have associated processing within 200 ms post-stimulus onset to visual awareness (Koivisto and Grassini, 2016) and sensory processing (Loughrey et al., 2023; Luck, 2023). Higher theta synchronization in the ARHL group relative to controls could be related to hyperexcitability due to cross-modal sensory enhancement. This aligns with evidence from electrophysiological studies (Puschmann et al., 2014; Slade et al., 2020; Madashetty et al., 2024), including a recent study that examined neural correlates of visual working memory tasks in older adults with ARHL (Loughrey et al., 2023). Our theta finding in the ARHL group is consistent with a few other studies (Petersen et al., 2015; Griffiths et al., 2020) and furthermore adds to the rapidly emerging body of work showing that sensory and cognitive processing interact more than has been previously acknowledged (Rönnberg et al., 2022a,b).

Group differences in information processing based on value (interaction between group and value) were observed at later time points (400-500 ms). Specifically, the ARHL group had greater theta synchronization for high- compared to low-value words. This was not observed for the NH controls. We did not expect this given that our previous studies have not found similar differences between theta synchronization for high- versus low-value words in cognitively healthy adults (Nguyen et al., 2020) and those with mild cognitive impairment (Nguyen et al., 2022). Whether these findings are specific to the ARHL group needs further validation. With theta synchronization linked to proactive control (as mentioned earlier), it could be that our participants with ARHL proactively engaged their top-down processes to strategically attend to and process high-value over low-value words as part of a compensatory neural mechanism as has been postulated by others (Loughrey et al., 2023). Studies have reported increased neural engagement of higher-order cognitive functions on non-auditory cognitive tasks in individuals with mild degrees of ARHL (Slade et al., 2020; Zhao et al., 2022; Loughrey et al., 2023). Given that theta is also linked to inhibitory control (Nigbur et al., 2011; Cavanagh and Frank, 2014; Cavanagh and Shackman, 2015), alternately, our findings might suggest that the ARHL group did the unexpected, i.e., used more neural resources linked to inhibitory control during the processing of high-value words (in other words, inhibited processing of high-value words), suggestive of maladaptive plasticity. However, our behavioral data does not support these findings, although further research, including modification of the current task, is necessary to truly connect our behavioral and ERSP findings. Regardless, our theta findings point toward potential cross-modal changes in those with mild ARHL, considering that the NH group did not demonstrate theta differentiation for high- versus low-value words. This aligns with emerging studies that have reported cross-modal plasticity in those with mild to moderate ARHL, especially in the visual cortical areas (Campbell and Sharma, 2014; Puschmann et al., 2014; Sharma and Glick, 2016; Ponticorvo et al., 2022). Further examination is needed to better delineate cross-modal reorganization in the context of visual strategic processing in ARHL. In general, our findings show that ARHL influences theta oscillations at earlier time points on a visual cognitive control task. Our findings align with other studies, both human (Heinrichs-Graham et al., 2022; Ryan et al., 2023) and animal (Johne et al., 2022), which have shown that hearing loss modulates oscillatory activity in the theta band. However, adequate comparison of our findings with others is difficult since we used a visual task while most have examined theta activity in relation to auditory tasks, primarily SiN recognition (Doelling et al., 2014; Hyafil et al., 2015; Etard and Reichenbach, 2019).

Some similarities were observed in both groups in strategic processing in the alpha band between 700 and 800 ms at the centroparietal electrode cluster. As hypothesized, both ARHL and NH groups had greater alpha desynchronization for high- than low-value words suggesting similar alpha modulation for processing information of varying values (i.e., high vs. low) in later time points. Alpha desynchronization in parietal regions is considered to reflect the engagement of attentional processes during selective attention (Foxe and Snyder, 2011; Deng et al., 2019; Trajkovic et al., 2023). The effect size of these alpha findings (more alpha desynchronization for high- than low-value words) was marginally larger in size within the NH group (Cohens’ d = 1.2) relative to the ARHL group (Cohen’s d = 1.12), suggesting a more robust modulation in the control group. We also found some similarities in our groups in theta oscillations. Greater frontal theta synchronization was observed for low-value relative to high-value words between 200 and 300 ms, and 700–1,000 ms. This suggests that both ARHL and NH groups strategically allocate more cognitive control resources to inhibit the processing of low-value information to prioritize the processing of high-value information, and these findings are consistent with our hypotheses and with our previous studies that used the same task in cognitively healthy (Nguyen et al., 2019, 2020) and cognitively impaired (Nguyen et al., 2022) populations. Both groups also showed greater alpha desynchronization for high- versus low-value words, between 400 and 1,000 ms in the centroparietal electrode cluster and between 200–300 ms and 600–1,000 ms in the parietal cluster, indicating greater attentional allocation for processing the high-value versus low-value words. The similarities between ARHL and NH groups, especially during the later time windows (700 ms and beyond) is similar to the findings of ERP studies involving P3 and late positive potential (LPP) on visual paradigms (Loughrey et al., 2023; Zhao et al., 2023). Perhaps, by the later stages of processing, those with ARHL are able to “catch up” to their age-matched NH peers by utilizing compensatory neurocognitive resources.

This study has several limitations. We analyzed ERSPs from a subset of electrodes that were defined *a priori* based on past literature. Future studies that use data-driven approaches, such as principal component analyses, would be useful to corroborate findings and advance knowledge related to neural oscillatory changes in older adults with mild ARHL. Future studies should examine ERSP changes with varying measures (e.g., power, phase coherence), as well as utilize complementary techniques such as source localization with EEG or functional magnetic resonance imaging to further unravel the neural mechanisms that underlie strategic processing in this population. Additionally, our study had a small sample size, and replicability from future studies is critical before the generalization of results. Finally, we did not develop the value-directed strategic processing task to distinguish between the processes of encoding, storage, and retrieval, and their examination is critical to understanding changes in value-directed strategic processing in ARHL.

In summary, the current study examined ERSPs underlying visual value-directed strategic processing in older adults with mild ARHL relative to NH controls of comparable age and education. Behavioral data revealed observable differences in total recall of words and total points earned, which points towards cognitive changes in those with ARHL. Changes in theta band were observed during early time periods in ARHL, but these were not specific to value-based processing, indicating more general changes in proactive control and cross-modal sensory enhancement. During the mid-time points (400–500 ms), the ARHL group showed greater theta modulations for high-value compared to low-value information, while these differences were not significant in controls. This might indicate adaptive changes in those with ARHL during strategic processing. In later time widows (after 700 ms), theta and alpha modulations were similar in both groups, perhaps indicating that the ARHL group does “catch up” to the NH controls. Our study adds to the emerging body of work on neural oscillatory changes underlying a cognitive control task in individuals with ARHL. Given that neural changes typically precede behavioral changes, these ERSPs can be used to assess the benefits of intervention (e.g., hearing aids, auditory and cognitive intervention programs) before behavioral changes are observable, especially given that our sample included individuals with untreated mild ARHL.

## Data availability statement

The original contributions presented in the study are included in the article/Supplementary material, further inquiries can be directed to the corresponding author.

## Ethics statement

The studies involving humans were approved by Institutional Review Board of the University of Illinois Urbana-Champaign (protocol # 17067). The studies were conducted in accordance with the local legislation and institutional requirements. The participants provided their written informed consent to participate in this study.

## Author contributions

SS: Data curation, Formal analysis, Investigation, Methodology, Project administration, Visualization, Writing – original draft, Writing – review & editing. SJ: Visualization, Writing – review & editing. RM: Conceptualization, Funding acquisition, Methodology, Resources, Supervision, Writing – review & editing.
