# Supplementary figures and images for: Alpha and theta oscillations on a visual strategic processing task in age-related hearing loss
Source: Front Neurosci. 2024 Jul 16;18:1382613. doi: 10.3389/fnins.2024.1382613 (PMC11289776; doi:10.3389/fnins.2024.1382613)

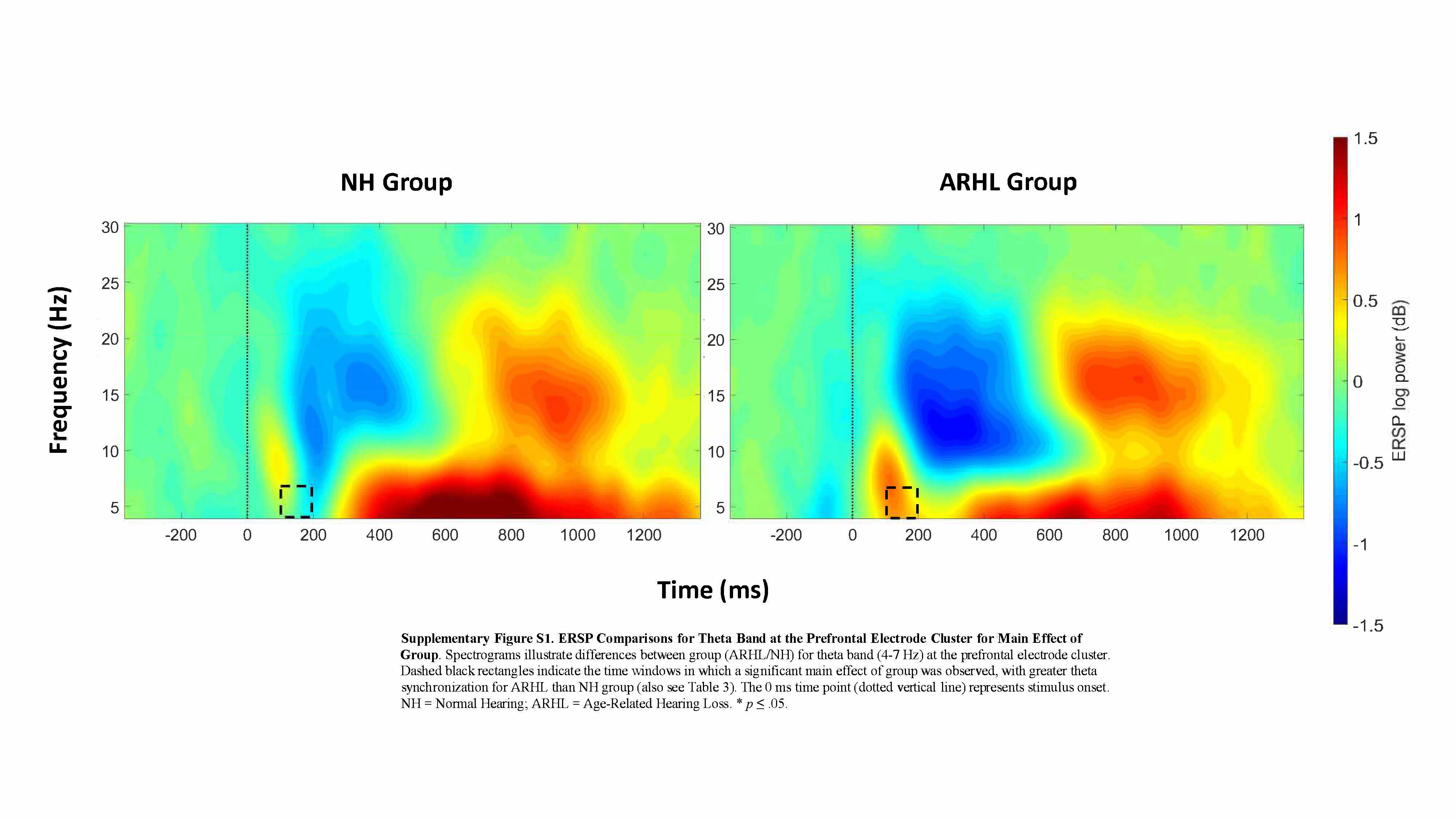

Supplement: Supplementary file 1 [file Image_1.jpg]

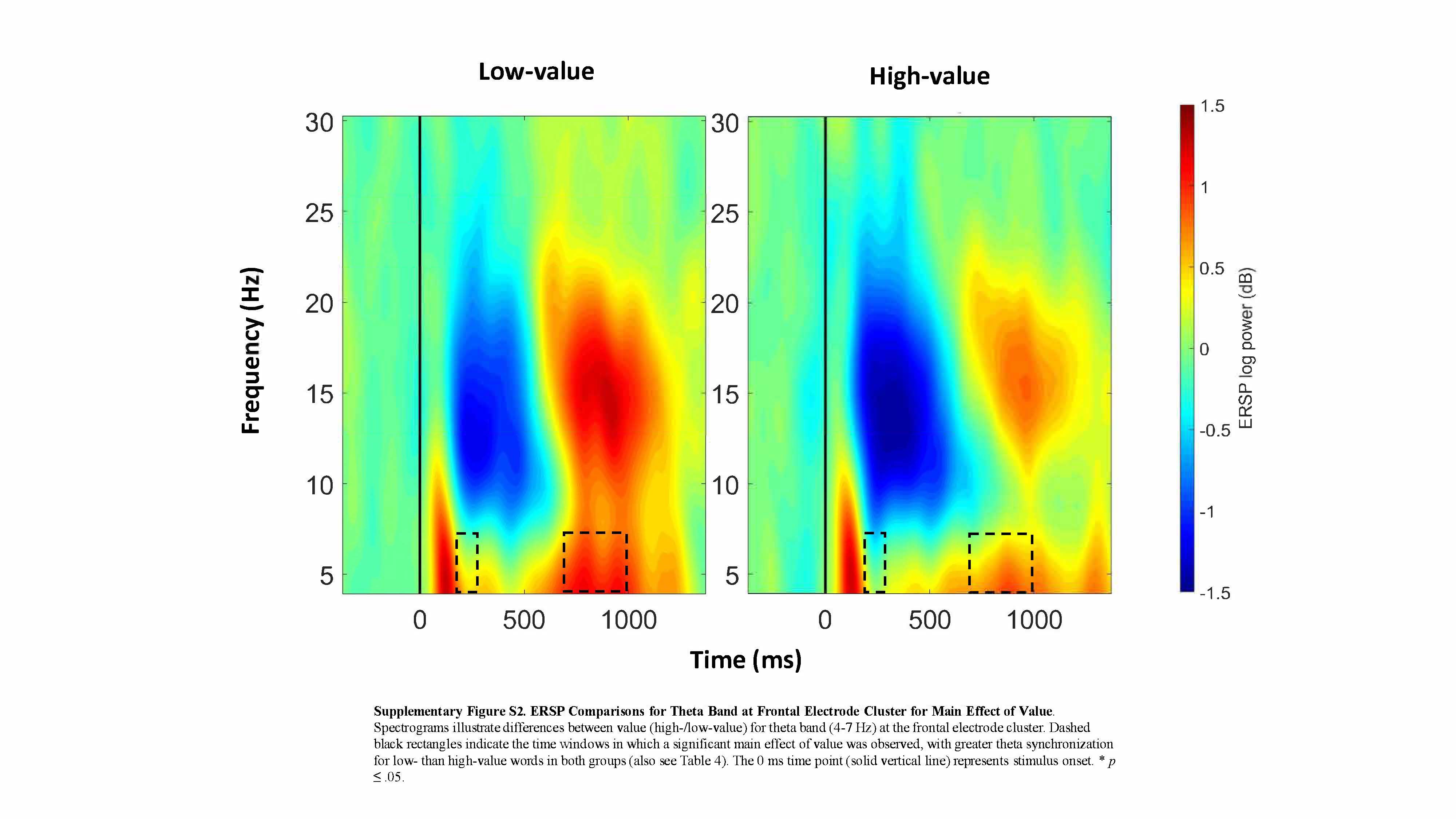

Supplement: Supplementary file 2 [file Image_2.jpg]

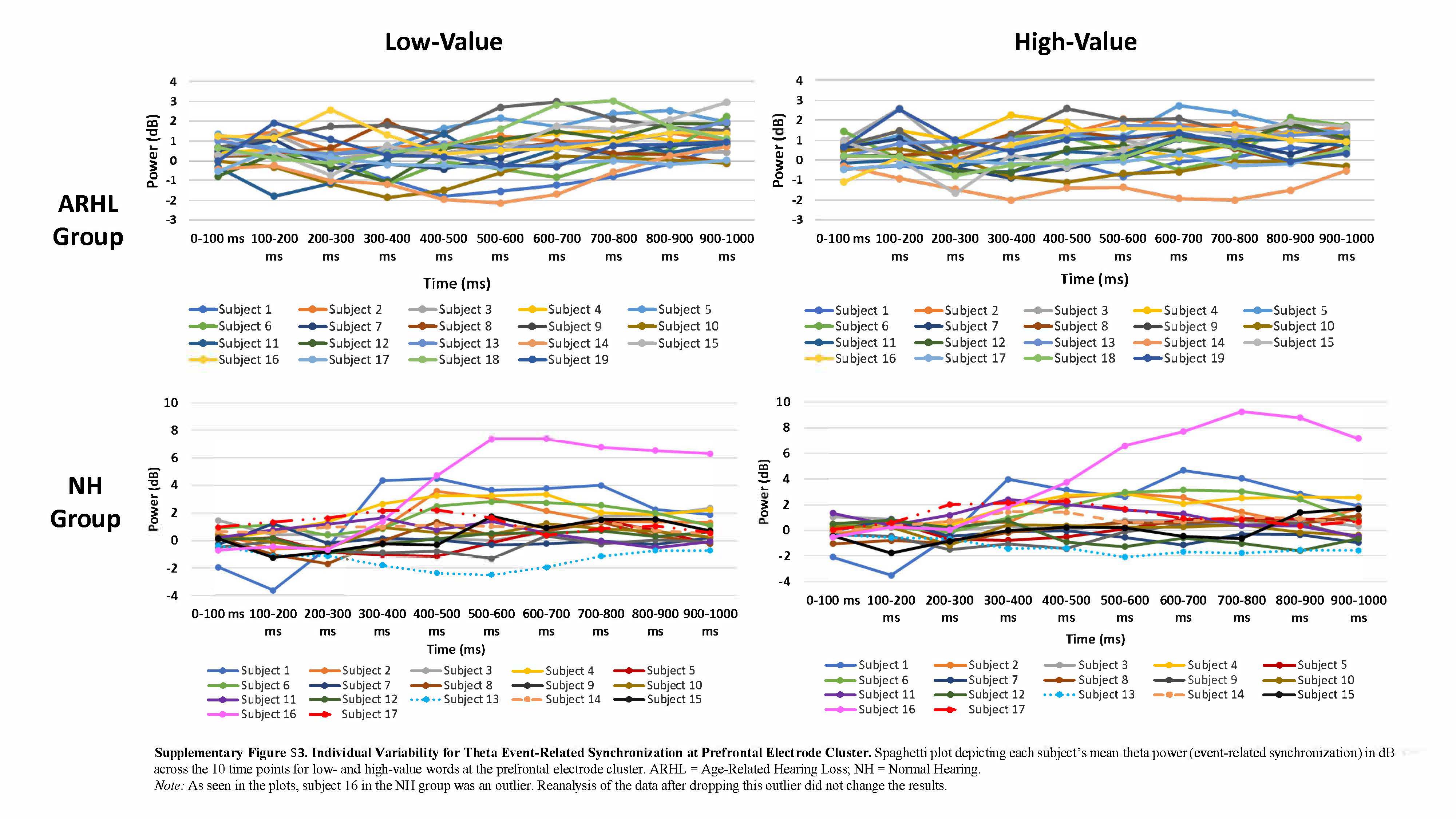

Supplement: Supplementary file 3 [file Image_3.jpg]

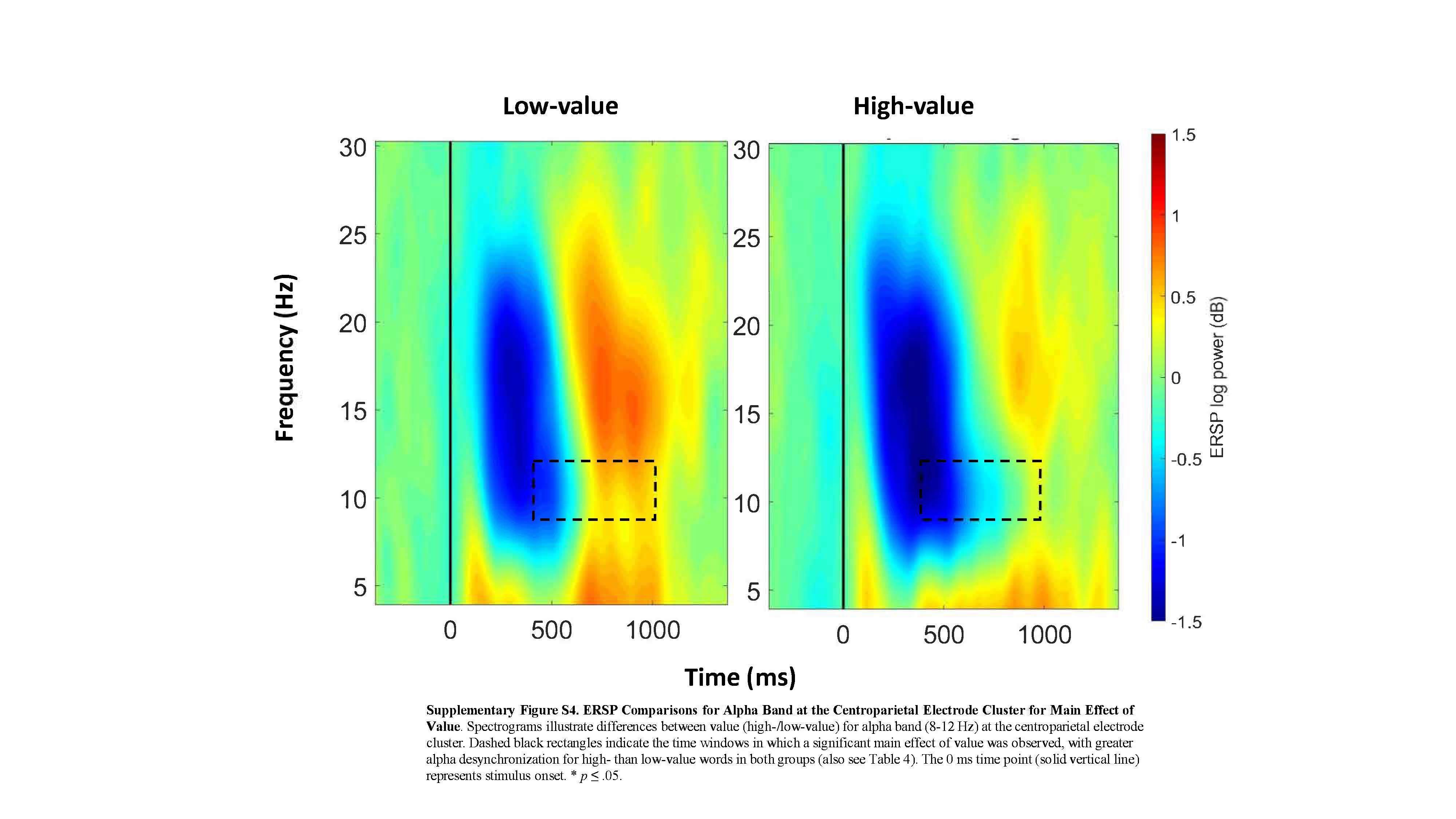

Supplement: Supplementary file 4 [file Image_4.jpg]

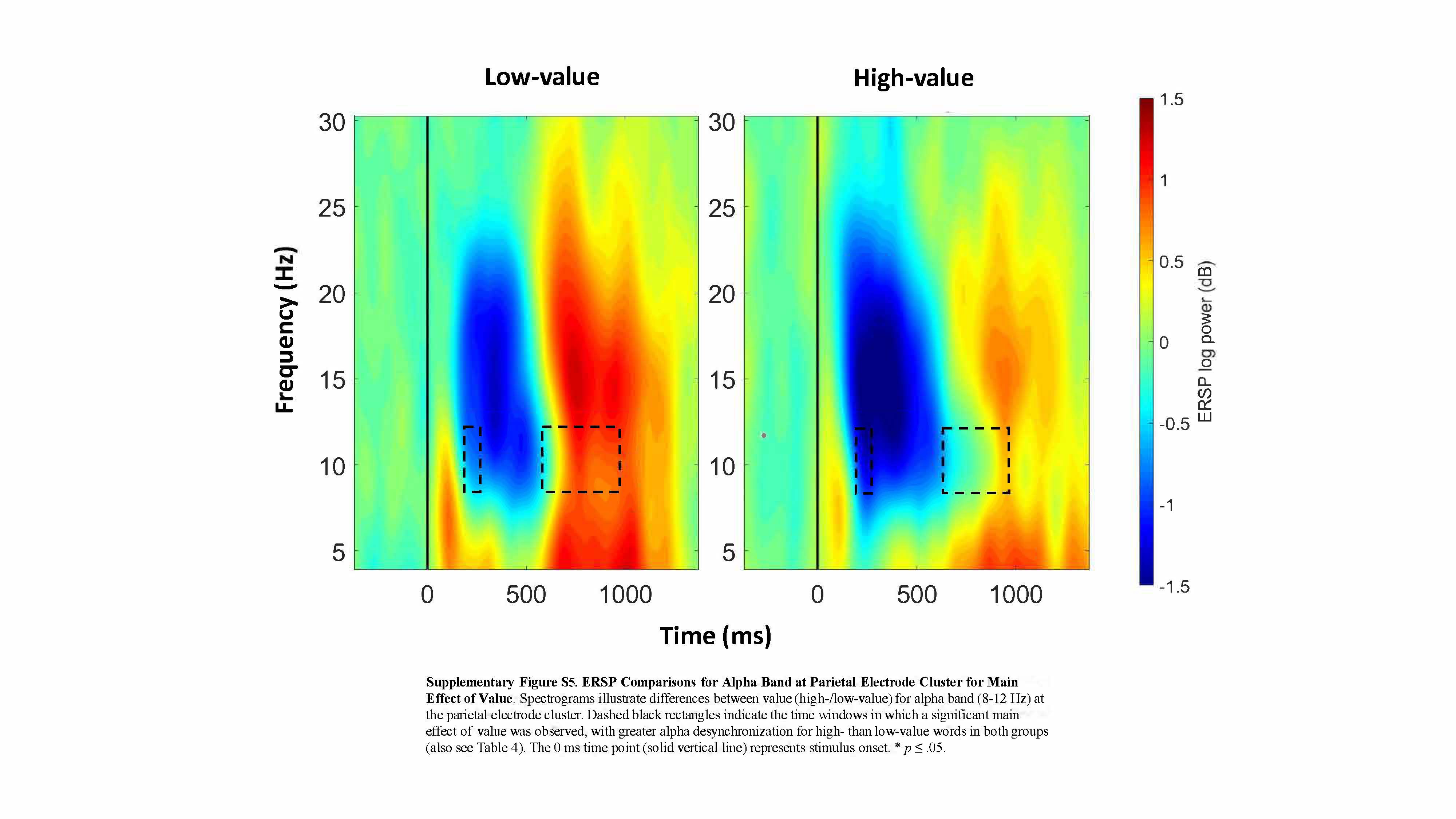

Supplement: Supplementary file 5 [file Image_5.jpg]

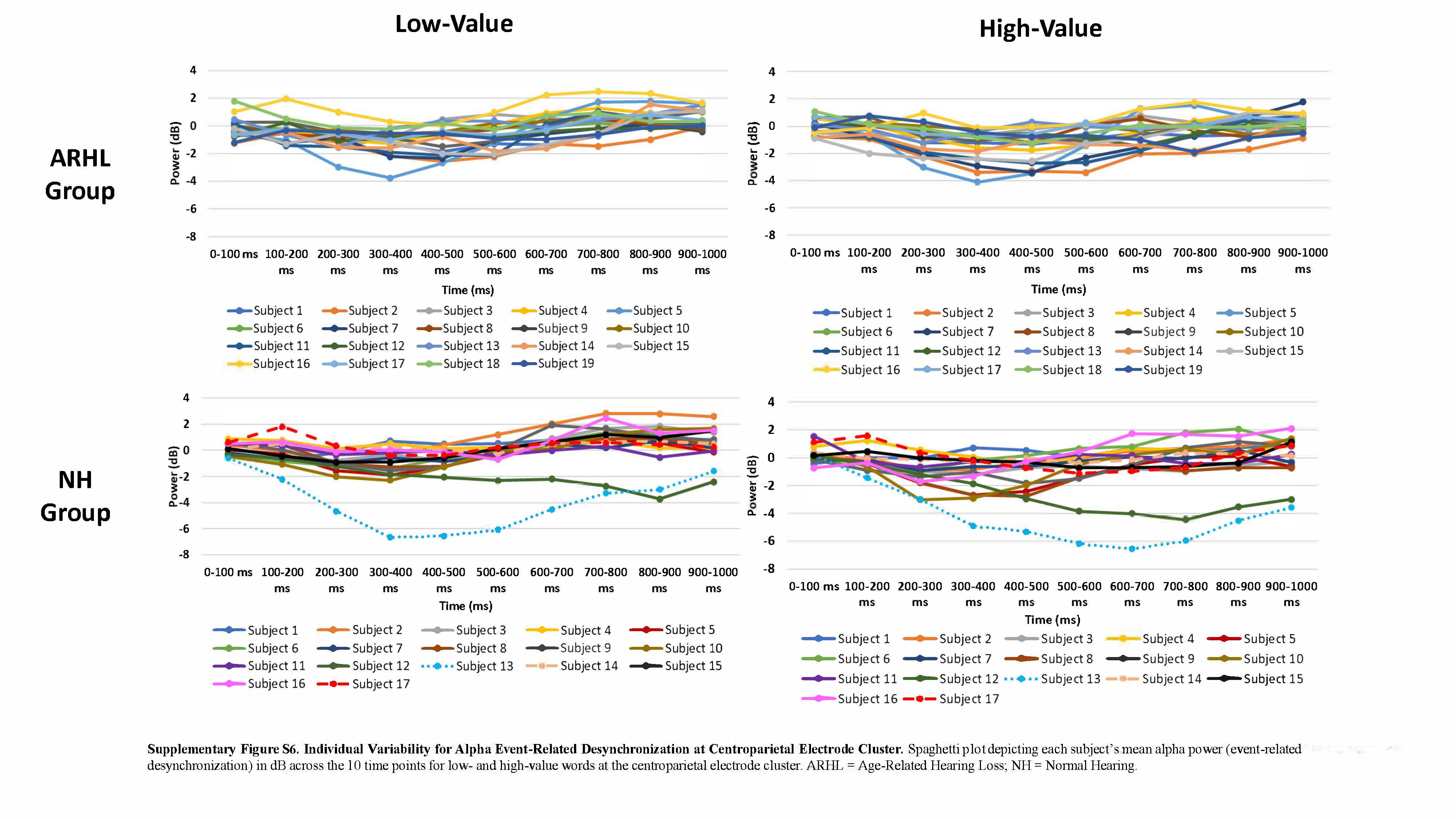

Supplement: Supplementary file 6 [file Image_6.jpg]
